# Lice infestation and diversity in turkeys (*Meleagris gallopavo*) in the Special Region of Yogyakarta and Central Java, Indonesia

**DOI:** 10.14202/vetworld.2020.782-788

**Published:** 2020-04-25

**Authors:** Joko Prastowo, Dwi Priyowidodo, Wisnu Nurcahyo, Defriana Lutfi Chusnaifah, Lu’lu’ Sahara Wusahaningtyas, Lintang Winantya Firdausy, Ana Sahara

**Affiliations:** 1 Department of Parasitology, Faculty of Veterinary Medicine, Universitas Gadjah Mada, Yogyakarta 55281, Indonesia; 2 Sains Veteriner Magister Program, Faculty of Veterinary Medicine, Universitas Gadjah Mada, Yogyakarta 55281, Indonesia

**Keywords:** central java, lice, *Phthiraptera*, turkey, Yogyakarta

## Abstract

**Background and Aim:**

Biting lice (*Phthiraptera*: *Amblycera* and *Ischnocera*) are ectoparasites that play important roles in the transmission of disease agents that infect turkeys and impact turkey productivity. This study aimed to determine the diversity of lice that infest turkeys in the Central Java Province and the Special Region of Yogyakarta, Indonesia.

**Materials and Methods:**

Lice sampling was conducted at 16 different locations from April 2019 to June 2019 in turkeys aged 4 months to 2 years. The samples were stored in 70% alcohol and were identified using avian louse keys. The morphology of the specimens was macroscopically and microscopically evaluated, and the resulting data were descriptively and qualitatively analyzed.

**Results:**

A total of 2505 lice were collected, and two families and five genera of lice were identified. Three lice genus members of the Philopteridae family (*Lipeurus*, *Oxylipeurus*, and *Chelopistes*) and two genera of the Menoponidae family (*Colpocephalum* and *Menacanthus*) were identified. *Lipeurus* was the most frequently identified genera in turkeys, whereas *Menacanthus* was the most rarely identified one. The White Holland breed had the highest number of lice infestations, whereas the Jersey Buff breed exhibited the highest diversity of lice genera. The average number of lice infestations was higher in male turkeys than in female turkeys.

**Conclusion:**

The occurrence of ectoparasites in domestic turkeys indicates that the existence and diversity of lice genera in the study location can be influenced by turkey type, turkey maintenance system, enclosure sanitation measures, lack of strategic ectoparasite control, and environmental factors.

## Introduction

Turkeys are a large group of birds found in North American forests, and they belong to the *Meleagris* genus [1]. *Meleagris gallopavo* or wild turkey is the ancestor of domestic turkeys that are found worldwide, including in Indonesia. Turkeys in Indonesia are known as “chicken-turkeys” because their body shape is similar to that of chickens. However, turkeys have a specific tail and snood shape compared with chickens. In addition, the turkey’s head is equipped with a throat wattle and caruncles [2]. Besides broilers, turkeys have a major contribution as the largest source of poultry meat. Although their egg production is very low, the meat produced from them can reach up to 20 kg/head. The global demand for turkey had increased each year until 2009, wherein turkey meat accounted for 5.8% of the world’s poultry meat commodity, with a production volume reaching 5.3 million tons [3]. Turkeys have several advantages compared with other animals, such as delicious taste, high protein content, and low fat and cholesterol contents [2]. The maintenance of broilers is fairly easy and does not require special care; moreover, broilers are more resistant to several diseases, including Marek’s disease and infectious bronchitis [3]. The benefits of raising turkey are more than those of raising native chickens, including a lower operational cost and a higher market demand [4,5]. Turkey is a type of poultry that began to develop in Indonesia in the last 10 years ; however, turkey meat is difficult to obtain to date because turkey population remains smaller than native chicken or broiler population. Furthermore, the number of turkey breeders in Indonesia remains low because few Indonesian people know how to rear turkeys. The maintenance of turkeys in Indonesia generally involves a semi-intensive system, which is divided into homogeneous and heterogeneous populations.

Raising turkeys also have challenges such as disease attacks and ectoparasite infestations. Several types of lice (*Phthiraptera*: *Amblycera*, *Ischnocera*) play important roles in transmitting various disease agents such as bacteria, viruses, and fungi [6]. These lice are located in several parts of the body, including the head, wings, neck, and abdomen [7]. The presence of ectoparasites can cause stress, irritation, discomfort, and anemia, which can reduce egg production [7]. Lice infestation can affect the carcass, appearance, and conformation of a turkey’s body and is also associated with hygiene and sanitation problems in turkey maintenance. Ectoparasite infestations in turkeys have been reported in several Western states; these ectoparasites include *Goniocotes gigas*, *Chelopistes meleagridis*, *Oxylipeurus polytrapezius*, *G. gallinae*, *Menacanthus stramineus*, and *Lipeurus lawrensis tropicalis* [8].

To the best of our knowledge, no study has reported data on lice infestation in turkeys in Indonesia. Therefore, the present study aimed to identify the diversity and occurrence rates of lice in infested turkeys in the Central Java Province and the Special Region of Yogyakarta, Indonesia.

## Materials and Methods

### Ethical approval

This study was based on the lice sample collection only, and ethical approval was not necessary.

### Lice collection

**Figure-1:**
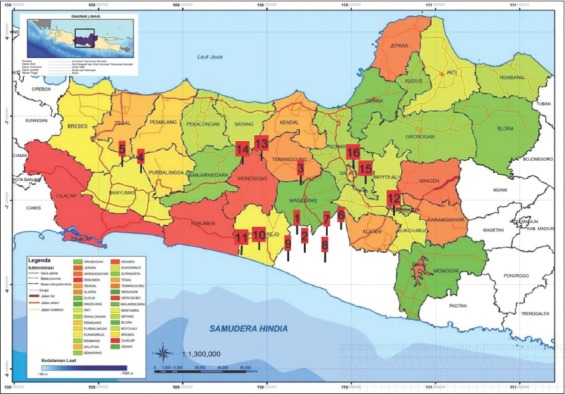
Location of turkey infestation sample collection in Central Java and D. I. Yogyakarta; (1) Minggir; (2) Moyudan; (3) Magelang; (4) Purwokerto (location 1); (5) Purwokerto (location 2); (6) Sindon; (7) Ngemplak; (8) Bantul; (9) Kulon Progo; (10) Purworejo (location 1); (11) Purworejo (location 2); (12) Sukoharjo; (13) Wonosobo (location 1); (14) Wonosobo (location2); (15) Salatiga (location1); (16) Salatiga (location 2). [Source: http://appejawa.navperencanaan.com/peta/viewmap?prov_code=jateng].

From April 2019 to June 2019, lice were collected from male to female turkeys aged 4 months to 2 years from 16 different locations in the Central Java Province and the Special Region of Yogyakarta, Indonesia (Figure-1). The lice on the surface of the turkey’s body were carefully removed using tweezers or thumb forceps. They were stored in glass bottles comprising 70% alcohol; the glass bottles were labeled with the location of collection, sex of the turkey, age of the turkey, and date of sampling.

### Morphological identification

Lice specimens were washed in distilled water and stored in 10% KOH for 24 h. Specimens that had been cleaned and transparent were washed again in distilled water and dehydrated in stages using 70%, 80%, and 95% alcohol for 20 min each. The dehydrated lice were placed in a mixture of 95% alcohol and clove oil for 3 min. The thin specimens were placed on glass slides using Canada balsam and dried in an incubator at 60°C for 7 days. Lice preparations were observed using an Olympus BX 51 microscope equipped with an Olympus DP 12 Digital camera and identified using previously reported louse keys; Menacanthus and Colpocephalum [9,10] and Chelopistes, Lipeurus, and Oxylipeurus [11].

## Results

A total of 2505 lice were collected from 29 turkeys of six different breeds located at 16 different locations. Based on the results of microscopic examinations, five lice genera, i.e., *Lipeurus*, *Oxylipeurus*, *Chelopistes*, *Menacanthus*, and *Colpocephalum*, were identified. The proportion of each genus and their distribution in breeds at different locations is shown in Table-1. The results of this study were descriptively and qualitatively analyzed because of the limited collection time. The highest number of lice infestations was found in turkeys from Salatiga 2 (17.13%), whereas the lowest number of lice infestations was found in turkeys from Kulon Progo (0.84%).

**Table-1:** The relative abundance and distribution of turkey’s lice genus in various area.

Location	Management system	Sex/No.	Breed	Lice genus (%)					Total per sex	Total (%)

				*Menacanthus*	*Lipeurus*	*Oxylipeurus*	*Colpocephalum*	*Chelopistes*		
Puton, Minggir^+^	Heterogen	M/1	B				44 (64.7)		44	68 (2.71)
		F/1	RP				24 (35.3)		24	
Moyudan^+^	Heterogen	M/1	WH		1 (4.1)	2 (8.3)	4 (16.7)	5 (20.8)	12	24 (0.96)
		F/1	RP		8 (33.3)	2 (8.3)	2 (8.3)		12	
Ngemplak^+^	Homogen	M/1	RP					23 (29.1)	23	79 (3.15)
		F/1	RP		31 (39.2)		25 (31.6)		66	
Bantul^+^	Heterogen	M/1	JB	2 (1.9)	12 (11.8)	31 (30.4)	19 (18.6)	38 (37.3)	102	102 (4.07)
Sindon^+^	Heterogen	M/1	B		34 (100)				34	34 (1.36)
KulonProgo^+^		M/1	RP					19 (90.5)	19	21 (0.84)
	Homogen	F/1	RP		2 (9.5)				2	
Magelang*	Homogen	M/1	BR		11 (29.7)			9 (24.3)	20	37 (1.5)
		F/1	BR		12 (32.4)			5 (13.5)	17	
Purwokerto 1*	Homogen	M/1	JB	3 (8.1)	8 (21.6)	6 (16.2)	2 (5.4)	10 (27)	29	37 (1.5)
		F/1	WH		6 (16.2)			2 (5.4)	8	
Purwokerto 2*	Heterogen	M/1	N					94 (63.5)	94	148 (5.9)
		F/1	N			39 (26.4)		15 (10.1)	54	
Purworejo 1*	Heterogen	M/1	N			15 (6.4)	55 (23.3)	79 (33.5)	149	236 (9.42)
		F/1	N		39 (16.5)	12 (5.1)	6 (2.5)	30 (12.7)	75	
Purworejo 2*	Heterogen	M/1	N			92 (46.5)		72 (36.4)	164	198 (7.9)
		F/1	N			34 (17.2)			34	
Sukoharjo*	Heterogen	M/1	WH		21 (5.2)	234 (58.2)		37 (9.2)	292	402 (16.05)
		F/1	N			69 (17.2)		41 (10.2)	110	
Wonosobo 1*	Homogen	F/1	WH		100 (100)				100	100 (3.99)
Wonosobo 2*	Homogen	M/1	B				111 (85.4)		111	130 (5.2)
		F/1	B		5 (3.8)	6 (4.6)	3 (2.3)	5 (3.8)	19	
Salatiga 1*	Heterogen	F/1	B		189 (44.1)				189	429 (17.13)
		M/1	WH	20 (4.7)	220 (51.3)				240	
Salatiga 2*	Homogen	F/1	WH		221 (48.0)				221	460 (18.36)
		M/1	B	32 (7.0)	207 (45.0)				239	
Total		F: 14 M: 15		57 (2.28)	1127 (44.99)	542 (21.64)	295 (11.78)	484 (19.32)	M=1572 F=931	2505 M=62.75 F=37.17

* =Central Java, +=Yogyakarta, B=Bronze, RP=Royal palm, WH=White Holland, JB=Jersey buff, BR=Bourbon red, N=Narragansett

**Figure-2:**
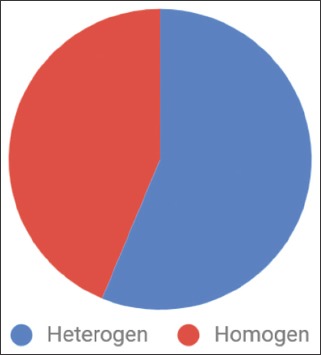
Management system of turkeys.

The diversity of lice genera varied in each turkey. All the domesticated turkeys examined for the presence of ectoparasites were found to be infested with one or more genera. In the present study, six turkey breeds, i.e., Bronze, Royal Palm, White Holland, Jersey Buff, Bourbon Red, and Narragansett, were maintained more heterogeneously than homogeneously (Figure-2). The number and diversity of lice infestations varied among breeds. The White Holland breed had the highest total number of lice on each turkey, whereas infestation events occurred most frequently in the Narragansett breed. The highest diversity of lice genera was found in the Jersey Buff breed. The species that infested the Jersey Buff turkeys comprised five genera, i.e., *Lipeurus*, *Oxylipeurus*, *Chelopistes*, *Menacanthus*, and *Colpocephalum*. The number and diversity of lice genera that infested male turkeys were more than those that infested female turkeys. Five ectoparasites were found in male turkeys from Purwokerto to Bantul. Furthermore, lice infestations were more common in male turkeys (62.75%).

The rate of infestation and the distribution of each lice genus varied at each location (Table-2). Among the five different types of lice genera identified, *Lipeurus* was the most distributed one with the highest number of infestation in turkeys. *Lipeurus* infestations were found at 13 of the 16 sampling locations, with an infestation rate of 62.07%. The rarest lice genus found at the 16 locations were *Menacanthus*, which was found only at four locations, i.e., Bantul, Purwokerto 1, Salatiga 1, and Salatiga 2, with an infestation rate of 13.79%.

**Table-2:** Number of turkey infested base on genera and sites of distribution.

Lice genus	Number of Turkey (%)	Sampling sites
*Menacanthus*	4 (13.79)	4
Lipeurus	18 (62.07)	13
Oxylipeurus	12 (41.38)	8
Colpocephalum	11 (37.93)	7
Chelopistes	16 (55.17)	11

**Figure-3:**
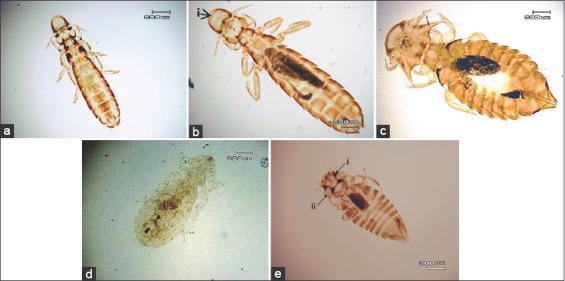
Lice genus identified in turkey (a) *Lipeurus* sp.; (b) *Oxylipeurus* sp., chitinized processes (i); (c) *Chelopistes* spp.; (d) *Menacanthus* sp.; (e) *Colpocephalum* spp., strong occipital nodi (i) and head with lateral notch (ii).

*Lipeurus* was found to have a long body shape with a pair of antennae that were visible and located beside the head. The head of *Lipeurus* had a rounded outer edge and comprised temporal lobes, and in addition to the legs, two small tarsal claws were found in each *Lipeurus* (Figure-3a). *Oxylipeurus* had the same characteristics as *Lipeurus*, except that the head of *Oxylipeurus* was equipped with chitinized processes (Figure-3b).

*Chelopistes* were found to have specific morphological characteristics; their heads were curved and extended from the temporal lobe toward the caudal direction, with the tip forming a long stylet-like process. Each tarsal of *Chelopistes* was equipped with two claws (Figure-3c).

*Menacanthus* and *Colpocephalum* had antennae that were not strongly clubbed and were partially or completely located under the head, and they had two claws in the tarsus. *Menacanthus* had a parabolic circular head and an elongated oval-shaped body, whereas *Colpocephalum* had a triangular head complemented by occipital nodes and lateral notches and an oval-shaped body that was quite small (Figure-3d and e).

## Discussion

According to the American Poultry Association [12], there are eight types of turkey breeds – Beltsville Small White, Black Spanish, Bourbon Red, Bronze, Narragansett, Royal Palm, Blue Slate, and White Holland. In the present study, White Holland, Bronze, Royal Palm, Narragansett, Jersey, and Bourbon Red breeds were obtained, accounting for six of the eight turkey breeds found worldwide. Moreover, the number and diversity of lice varied by turkey breed as well as turkey sex, which are contradictory to that reported by Saxena *et al*. [13,14] and Singh *et al*. [15,16] who reported that the color of the feathers and the sex of poultry have no effects on the incidence of infestation. Majood *et al*. [17] and Santa [18] reported that the proportion of lice infestation in turkeys was mainly influenced by external factors, such as environmental conditions of the cage and the maintenance system. It has been reported that intensive systems will reduce the risk of high types of ectoparasites infesting the birds [19]. A bird can be infested with more than one species of ectoparasites because the ectoparasites could transmit from one bird species to another by direct contact in the free-range system [20,21]. The poultry rearing system in Indonesia is divided into three systems: The extensive traditional system, the semi-extensive system, and the intensive system [22,23]. The semi-extensive system with a heterogeneous population is generally used in Indonesia for turkey maintenance. Therefore, the number and variety of lice in both the heterogeneous and homogeneous maintenance populations were relatively similar in the present study. Furthermore, climate, temperature, uncontrolled nutrition, and sanitation also affect the incidence of lice infestation in birds or poultry [17,18]. Indonesia is a tropical region with two seasons and relatively warm average annual temperatures at approximately 26–36°C. This temperature is ideal for the breeding and growth of various insects, including the lice that infest turkeys.

Morphological identification results of lice were obtained from two families, i.e., Menoponidae and Philopteridae. Both of these families are feed in bird, hence they have two claws in each tarsus. Beside the menoponids has a broad and blunt-shape head with antennae that located under the head, it also has separate mesonotum and metanotum in their thorax. In addition, the first spiracle can be found on the border between the thorax and the abdomen. Philopteridae have five filiform antennae segments, with the male louse antennae being larger than the female louse antennae [9,10].

The Menoponidae found in the present study comprised the *Colpocephalum* and *Menacanthus* genera, which are often found in the barbs of flight feathers in turkeys. *Colpocephalum* and *Menacanthus* are distinguishable by their head shapes. *Colpocephalum* has a triangular head equipped with occipital nodes and lateral notches and an oval-shaped body that is quite small. However, *Menacanthus* has a parabolic circular head that is complemented by a pair of spines in the ventral section of the head without a microconidium region; it also has an elongated oval-shaped body [9,10].

There are approximately 135 species of *Colpocephalum* that is generally found in birds of the Cuculiformes, Ciconiiformes, *Columbiformes*, Falconiformes, *Galliformes*, *Gruiformes*, *Passeriformes*, *Pelecaniformes*, *Piciformes*, *Psittaciformes*, and *Strigiformes* families [10]. Turkeys belong to the Galliformes family. The incidence of *Colpocephalum* infestations has been reported in wild birds such as *Buteo rufinus* and *B. buteo* [24]; however, to the best of our knowledge, *Colpocephalum* infestations have never been reported in turkeys, especially in those found in Southeast Asia.

*Menacanthus* can be found in birds of the *Coraciiformes*, Cuculiformes, Galliformes, Passeriformes, *Piciformes*, and Tinamiformes Price families [10]. *Menacanthus* infestations have often been reported in turkeys in Southeast Asia and other parts of the world, including Thailand [25], Malaysia [26], the USA [27], Iran [28], Iraq [29], Nigeria [8], and Ukraine [30]. Two species of *Menacanthus* have been reported to infest turkeys in Southeast Asia, i.e., *M. stramineus* in Thailand [25] and *M. pallidulus* in Malaysia [26].

The members of the Philopteridae family found in the present study included *Lipeurus*, *Oxylipeurus*, and *Chelopistes*. *Lipeurus* was found to have a long body shape with visible antennae located next to the head. The head of *Lipeurus* has a rounded outer edge and comprised temporal lobes, and in addition to each leg, two small tarsal claws are found in *Lipeurus*. The *Oxylipeurus* obtained in the present study were found to have the same characteristics as *Lipeurus*, except for the head, which was equipped with chitinized processes. Male *Oxylipeurus* have a subgenital plate with a medioposterior process [10,11]. *Lipeurus* and *Oxylipeurus* are found more often in the rachis of flight and tail feathers and less often in those of contour feathers. *Lipeurus* can be found in turkeys in Nigeria [8] and Malaysia [26], whereas *Oxylipeurus* can be found in turkeys in the USA [27] and Iraq [29].

*Chelopistes* were found to have specific morphological characteristics: Their head is curved and extends from the temporal lobe toward the caudal direction, with the tip forming a long stylet-like process. In the present study, *Chelopistes* were found in the skin and body feather (body contour and semiplume aftershaft) of turkeys. *Chelopistes* reportedly infest ocellated turkeys (*Meleagris ocellata*) from South Mexico and Central America [7]. These parasite infestations can also be commonly found in the bodies of turkeys from the USA [27], Iran [28], Nigeria [8], Thailand [25], Filipina [31], and Malaysia [26]. Lice are the most specific hosts among all ectoparasites. Many species of chewing lice have been found on only one genus or species of host, whereas some species of chewing lice are less specific [32]. In this case, *Chelopistes* are specifically found in turkeys, whereas other genera found in the present study are often found in various other types of birds [25,26,33].

## Conclusion

This is the first study to determine the prevalence and species diversity of ectoparasites found in domestic turkeys in the Central Java Province and the Special Region of Yogyakarta, Indonesia. The occurrence of ectoparasites in domestic turkeys indicates that the existence and diversity of lice genera in the study location can be influenced by turkey type, turkey maintenance system, enclosure sanitation measures, lack of strategic ectoparasite control, and environmental factors. Further studies are warranted to evaluate their spatiotemporal pattern and analyze its direct impact on animals and humans in remote and agricultural regions of the country.

## Authors’ Contributions

The research was designed, managed, and supervised by AS. JP, AS, DP, and WN analyzed the samples, drafted and revised the manuscript. DLC, LSW, and LWF collected and processed the samples. All authors have read and approved the final manuscript.
